# Post-exposure Treatment with Anti-rabies VHH and Vaccine Significantly Improves Protection of Mice from Lethal Rabies Infection

**DOI:** 10.1371/journal.pntd.0004902

**Published:** 2016-08-02

**Authors:** Sanne Terryn, Aurélie Francart, Heidi Rommelaere, Catelijne Stortelers, Steven Van Gucht

**Affiliations:** 1 National Reference Centre of Rabies, Viral Diseases, Scientific Institute of Public Health (WIV-ISP), Brussels, Belgium; 2 Laboratory of Virology, Department of Virology, Parasitology and Immunology, Faculty of Veterinary Medicine, Ghent University, Ghent, Belgium; 3 Ablynx NV, Ghent, Belgium; Wistar Institute, UNITED STATES

## Abstract

Post-exposure prophylaxis (PEP) against rabies infection consists of a combination of passive immunisation with plasma-derived human or equine immune globulins and active immunisation with vaccine delivered shortly after exposure. Since anti-rabies immune globulins are expensive and scarce, there is a need for cheaper alternatives that can be produced more consistently. Previously, we generated potent virus-neutralising VHH, also called Nanobodies, against the rabies glycoprotein that are effectively preventing lethal disease in an *in vivo* mouse model. The VHH domain is the smallest antigen-binding functional fragment of camelid heavy chain-only antibodies that can be manufactured in microbial expression systems. In the current study we evaluated the efficacy of half-life extended anti-rabies VHH in combination with vaccine for PEP in an intranasal rabies infection model in mice. The PEP combination therapy of systemic anti-rabies VHH and intramuscular vaccine significantly delayed the onset of disease compared to treatment with anti-rabies VHH alone, prolonged median survival time (35 versus 14 days) and decreased mortality (60% versus 19% survival rate), when treated 24 hours after rabies virus challenge. Vaccine alone was unable to rescue mice from lethal disease. As reported also for immune globulins, some interference of anti-rabies VHH with the antigenicity of the vaccine was observed, but this did not impede the synergistic effect. Post exposure treatment with vaccine and human anti-rabies immune globulins was unable to protect mice from lethal challenge. Anti-rabies VHH and vaccine act synergistically to protect mice after rabies virus exposure, which further validates the possible use of anti-rabies VHH for rabies PEP.

## Author Summary

Rabies is an infectious disease causing 59,000 deaths and millions are exposed each year worldwide. Post-exposure prophylaxis (PEP) against rabies consists of a combination of passive (immune globulins) and active immunisation (vaccine) directly after viral exposure. Currently used plasma-derived anti-rabies immune globulins are expensive and scarce, urging the development of alternatives. Nanobodies or VHH are the smallest antigen-binding fragments of camelid heavy chain antibodies and are easy to produce with intrinsic good thermal stability and solubility. Combined treatment with anti-rabies VHH and vaccine gave significantly better protection than either compound alone in an intranasal rabies challenge model in mice, which validates the potential use of anti-rabies VHH as replacement of immune globulins in PEP.

## Introduction

Rabies virus ultimately causes an aggressive and lethal infection in the brain of humans and other mammals. Rabies virus is a model neurotropic RNA virus that belongs to the family *Rhabdoviridae*, *Genus Lyssavirus* [1;2]. The virus is transmitted through the saliva of an infected animal by biting or scratching. Once the virus enters peripheral nerves or neurons, it quickly replicates in the neuronal cytoplasm and progeny virus is transported through the neuronal network by crossing tight interneuronal synapses, eventually giving rise to encephalitis [3;4]. Each year, an estimated 59000 people die from rabies and about 29 million receive post-exposure prophylaxis (PEP) after close contact with a suspected animal [5].

Passive antibody therapy with anti-rabies immune globulins (RIG) plays a major role in rabies post-exposure prophylaxis after high risk exposure [6]. Together with thorough wound cleansing, it is the first line of defence against the virus, and prophylaxis without RIG is associated with treatment failure [7;8]. Pioneering studies on the effects of anti-rabies serum date back to the late 1800s and early 1900s, and since 1954 the World Health Organisation (WHO) recommends the use of RIG in combination with vaccination for rabies post-exposure prophylaxis [9]. Treatment with RIG and vaccine should be initiated as soon as possible after potential infection, with additional vaccine administrations in the following weeks to activate a full-blown and lasting immune response. Passive immunization with RIG serves to immediately neutralize the virus and close the gap between viral exposure and the vaccine-induced immune response [7]. In this regime, initial protection is offered by RIG, which is then gradually replaced by vaccine-induced antibodies mounted between day 0 and 7–14, providing continued protection to patients [10].

Rabies antibodies can be either from equine (ERIG) or human (HRIG) origin. Due to adverse effects, such as serum sickness, equine antibodies are now used under the form of pepsin-digested Fab fragments, but if available, HRIG is still preferred over ERIG [9]. The production of HRIG, however, requires sufficient numbers of immune donors and gives rise to the typical problems associated with biological products of human origin, such as the transmission of infectious agents [9]. The worldwide shortage and the high costs makes these products poorly available to developing countries, where rabies is endemic [7;9], the reason why the WHO recommends to develop alternatives [11].

VHH or Nanobodies (a trade-name by Ablynx) are the smallest functional fragments (15 kDa) of heavy chain-only antibodies naturally occurring in *Camelidae*, and represent the antigen-binding variable domain. By nature VHH are hydrophilic and do not require hydrophobic interactions with a light chain, which allows high solubility, physicochemical stability and high-yield production in *Escherichia coli*, yeast or mammalian expression systems. The single domain nature and the small size of VHH also allows for easy formatting by genetic fusion into multimeric and multispecific constructs [12–14].

Previously, we generated a potent neutralizing anti-rabies VHH recognising two epitopes on the rabies glycoprotein, fused to an anti-albumin VHH to extend its serum half-life (HLE). The Rab-E8/H7-ALB11 was able to neutralize the virus at picomolar doses [15]. Post exposure treatment with anti-rabies VHH at 24 hours after intranasal virus challenge could significantly delay disease onset in mice, and depending on the dose, could rescue part of the mice from lethal disease [15].

The main aim of this study was to examine whether the combined treatment with anti-rabies VHH and vaccine (Rabipur, Novartis) after exposure to rabies virus has added value compared to single treatment with either compound in the intranasal rabies virus challenge model, which is very well suited to study intervention strategies for prevention and prophylaxis [16]. Via the intranasal route the virus can directly access the brain via the olfactory epithelium, which results in a highly reproducible infection [17]. First disease signs appear at 7 days, which rapidly progress the following 2 days, requiring euthanasia at 8–9 days post inoculation (DPI). This model was recently also proposed as a valuable alternative to intracranial inoculation for rabies vaccine potency testing [18]. The typically short incubation period of this model (6.07 ± 0.59 days) is ideal to study the potentially beneficial effect of the combined passive (VHH) and active (vaccine) immunisation on disease outcome. Our results show that anti-rabies VHH and vaccine act synergistically to protect mice after rabies virus exposure, which further validates the possible use of anti-rabies VHH for rabies PEP.

## Materials and Methods

### VHH and antibodies

VHH directed against the rabies virus glycoprotein G were described previously [19]. Briefly, llamas were vaccinated using the inactivated rabies Human Diploid Cell Vaccine (HDCV, Sanofi, France) and RNA was extracted from peripheral blood lymphocytes. VHH genes were amplified from a cDNA library. Anti-rabies VHH were selected by panning phage libraries on plates coated with the native G protein. Multivalent VHH constructs were generated by the fusion of monovalent VHH into multimeric VHH constructs using flexible glycine-serine (GS) linkers [20]. In this study, we used the half-life extended VHH (HLE Rab-E8/H7-ALB11), containing two different VHH against the rabies virus spike protein and an anti-albumin VHH (ALB11) for half-life extension, and the non-HLE Rab-E8/H7 [15]. VHH was produced and kindly provided by Ablynx (Zwijnaarde, Belgium).

Human rabies immune globulins (HRIG) (Berirab, CSL Behring GmbH, Germany) are gammaglobulins purified from plasma of vaccinated human donors.

### Vaccine

Rabipur (Purified Chicken Embryo Cell Vaccine, Novartis, Belgium) was reconstituted according to the manufacturer’s instructions and was administered via intraperitoneal or intramuscular injection. The vaccine contains at least 2.5 antigenic units (AU)/ml. It contains the inactivated Flury LEP strain produced on purified chick embryo cells.

### Rabies virus

Challenge Virus Standard (CVS)-11 is a virulent classical rabies virus obtained from the American Type Culture Collection (ATCC reference VR959) and was grown in baby hamster kidney (BHK)-21 cells. For virus inoculation in mice, a dose of 10^2.5^ 50% cell culture infectious doses (CCID_50_) was used.

### Mouse experiments and clinical follow-up

Six-to-eight weeks old female Swiss outbred mice (Charles River, France) were used. Mice were kept in filter top cages, water and feed provided *ad libitum* and exposed to a natural day/night light cycle. Intranasal (IN) inoculation procedures are described in detail by Rosseels *et al*. [16]. The intranasal inoculation of rabies virus is an excellent technique to study antiviral treatment in the brain, since it leaves the brain mechanically intact, in contrast to intracranial inoculation, and yields a highly reproducible brain infection and disease outcome with little variation in the median survival time. This inoculation route has been used before for the evaluation of post exposure prophylaxis of rabies in mice [21]. For intraperitoneal (IP) or intramuscular (IM) injections maximum volumes of respectively 1000 and 100 μl were respected (50 μl per site in case of IM injections). Prior to intramuscular or intranasal administrations, mice were briefly anesthetized using isoflurane gas (IsoFlo, Abbott laboratories Ltd., United Kingdom), as described by Rosseels *et al*. (2011) [16].

Three retro-orbital bleedings were performed under isoflurane anaesthesia during the 28 day immunization period.

Mice were observed daily for signs of disease until 35 days post virus inoculation. Mice develop a typical disease pattern, which progresses as follows: isolation from the group (score 1), slow/less vivid movement (score 2), paresis in paws (score 3), uncoordinated movement (score 4), absence of spontaneous movement (score 5), no response to stimuli (score 6) and the end-stage characterized by mice burying their heads in cage bedding and slow breathing (score 7). The score per mouse ranges thus from 0 (no disease) to 7 (severe nervous disease). Disease progression was represented by plotting the daily score in function of the days post inoculation (DPI). The incubation period was defined as the period between virus inoculation and the first appearance of disease signs. In our experience, mice with a disease score of 6 or more die within 24 hours. Therefore, mice were euthanized by cervical dislocation when they reached a score of ≥ 6. Results were expressed with Kaplan-Meier survival curves. Rabies virus infection in the brain was confirmed using real-time reverse transcriptase polymerase chain reaction (RT-qPCR) as described by Suin *et al*. [22], and by the fluorescent antigen test (FAT), performed according to the Manual of Diagnostic Tests and Vaccines for Terrestrial Animals (Office International des Epizooties, 2008).

### Determination of the viral load

The viral RNA load in the brain of mice was determined using RT-qPCR, as previously described [15;22]. Briefly, the brain was homogenized and RNA was extracted according to the manufacturer’s instructions (RNeasy kit, Qiagen, Hilden, Germany). Ribosomal 18S was used as a reference gene for standardization and delta cycle thresholds (Δ Cq) values were calculated using the following formula: Δ Cq = Cq_ref_−Cq, with Cq_ref_ equal to 45, the number of cycles in this program.

### Virus-neutralisation test

The virus-neutralizing titer of serum, antibody and VHH preparations was determined with the Rapid Fluorescent Focus Inhibition Test (RFFIT), according to the Manual of Diagnostic Tests and Vaccine for Terrestrial Animals (Office International des Epizooties, 2008). The neutralizing potency is expressed in international units (IU)/ml in reference to "The Second International Standard for Anti-Rabies Immunoglobulin", purchased from the United Kingdom National Institute for Biological Standards and Control.

### Statistical analysis

GraphPad Prism was used for statistical analyses of *in vivo* data. Differences in survival times were tested using the Log-Rank test with a Bonferroni post-test, differences in Δ Cq values were tested using a Student’s t-test after normalization to the house-keeping gene. Differences in antibody titers were also tested using a Student’s t-test.

### Ethics statement

All experimental procedures were approved by the Ethical Commission of the WIV-ISP and CODA-CERVA (advice number 070515–05) and were performed according to the EU Directive 2010/63/EU for animal experiments.

## Results

### 1. Pre-exposure vaccination

To validate the protective effect of rabies vaccine (Rabipur, Novartis) in the intranasal rabies mouse model, mice were vaccinated with two intramuscular vaccine doses (0.25 AU/mouse), with a 3-day interval, following the schedule also used later on for PEP. This vaccination schedule is schematically represented in Table 1. Mice received a viral challenge 25 days after the last vaccine, allowing sufficient time for the development of an immune response.

**Table 1:** Set-up of the pre-exposure vaccination experiment and interventions in different treatment groups.

Group	Intervention at day…			
	- 28	- 25	0	35
**Vaccine + VHH (n = 9)**	Vaccine + anti-rabies VHH	Vaccine	Virus challenge	End observation period—euthanasia
**Vaccine only (n = 10)**	Vaccine	Vaccine	Virus challenge	End observation period—euthanasia
**VHH only (n = 8)**	Anti-rabies VHH	Saline	Virus challenge	End observation period—euthanasia
**Saline (n = 10)**	Saline	Saline	Virus challenge	End observation period—euthanasia

**Fig 1:**
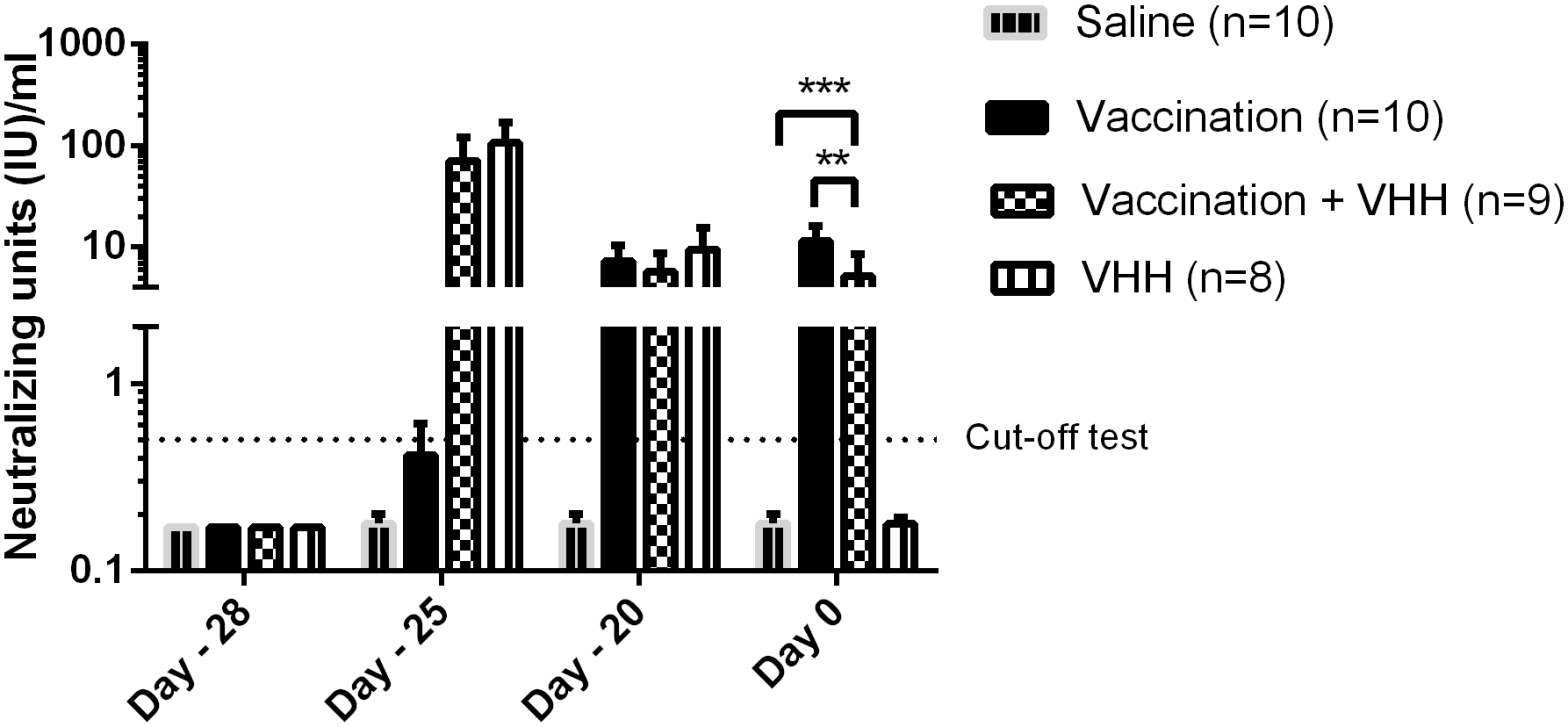
Rabies neutralizing activity in the blood as measured by RFFIT, following intramuscular (IM) vaccination at day -28 and day-25 either alone or in combination with anti-rabies VHH (Rab-E8-H7-ALB11, 1.5 mg/mouse) at day -28. Control groups consisted of mock treatment with saline (0.9% NaCl) or anti-rabies VHH only at day -28. Blood was collected at day -28 (prior to vaccination and VHH administration), day -25, day -20 and day 0 (= time of virus challenge). Both groups of mice that received anti-rabies VHH had high neutralizing titers at the early time points (day -25 and -20). Mice that received vaccine had neutralizing antibodies at day -20, which further increased to high levels at day 0. Mice treated with anti-rabies VHH only no longer had detectable VHH at day 0. Antibody titers in the vaccine + VHH group were significantly lower than in the vaccine only group at day 0 (** p< 0.005, *** p< 0.001). Error bars represent the standard deviation.

The mounting of the humoral immune response in the blood after vaccination was monitored by assessing the rabies neutralization activity *in vitro* (RFFIT) in blood collected at different time points. In Fig 1, it is shown that mice that received the vaccine had detectable antibody titers eight days after the first dose (day -20), (mean 7.22 ± 3.28 IU/ml, range 3.73–12.62 IU/ml), which were well above the generally accepted protective threshold of 0.5 IU/ml. Antibody titers continued to increase until 28 days later (day 0, mean 11.47 ± 4.77 IU/ml, range 6.01–14.81 IU/ml).

To verify if the efficacy of the vaccine would be affected by the simultaneous administration of anti-rabies VHH, an interference phenomenon which is well known for anti-rabies immune globulins [23], a group of mice received besides the vaccine also a single dose of anti-rabies VHH in the same pre-exposure setting. In this regime, the first vaccination (day-28) was accompanied by anti-rabies VHH (Rab-E8/H7-ALB11) at a dose of 1.5 mg/mouse (corresponding to 60 mg/kg, 392600 IU/kg), at the moment of the first vaccination (day -28). Vaccine (IM) and VHH (IP) were administered at separate sites. As reference groups mice were treated with anti-rabies VHH alone, or left untreated. In mice, the half-life of the anti-albumin VHH is approximately 1.5 days, hence the anti-rabies VHH will be removed from the circulation at the moment of viral challenge.

Fig 1 shows that the rabies neutralization titers of mice that were injected with anti-rabies VHH, whether or not in combination with vaccination, were high 3 days after VHH administration (day -25, mean 88.28 ± 58.05 IU/ml, range 0.61–149.18 IU/ml). As expected, anti-rabies VHH titers rapidly declined over time with the clearance of the VHH from the blood (day -20, mean 9.43 ± 6.04 IU/ml, range 0.16–15.57 IU/ml) and no detectable titers (< 0.5 IU/ml) on day 0. Mice that received both vaccine and anti-rabies VHH had a mean titer of 5.69 ± 3.03 IU/ml (range 1.73–9.37 IU/ml) at day -20, similar to mice that received vaccine alone, while at day 0, antibody titers were significantly (p<0.005) lower in the vaccine + VHH group (mean 5.15 ± 3.38 IU/ml, range 0.37–10.03 IU/ml), compared to the vaccine only group.

**Fig 2:**
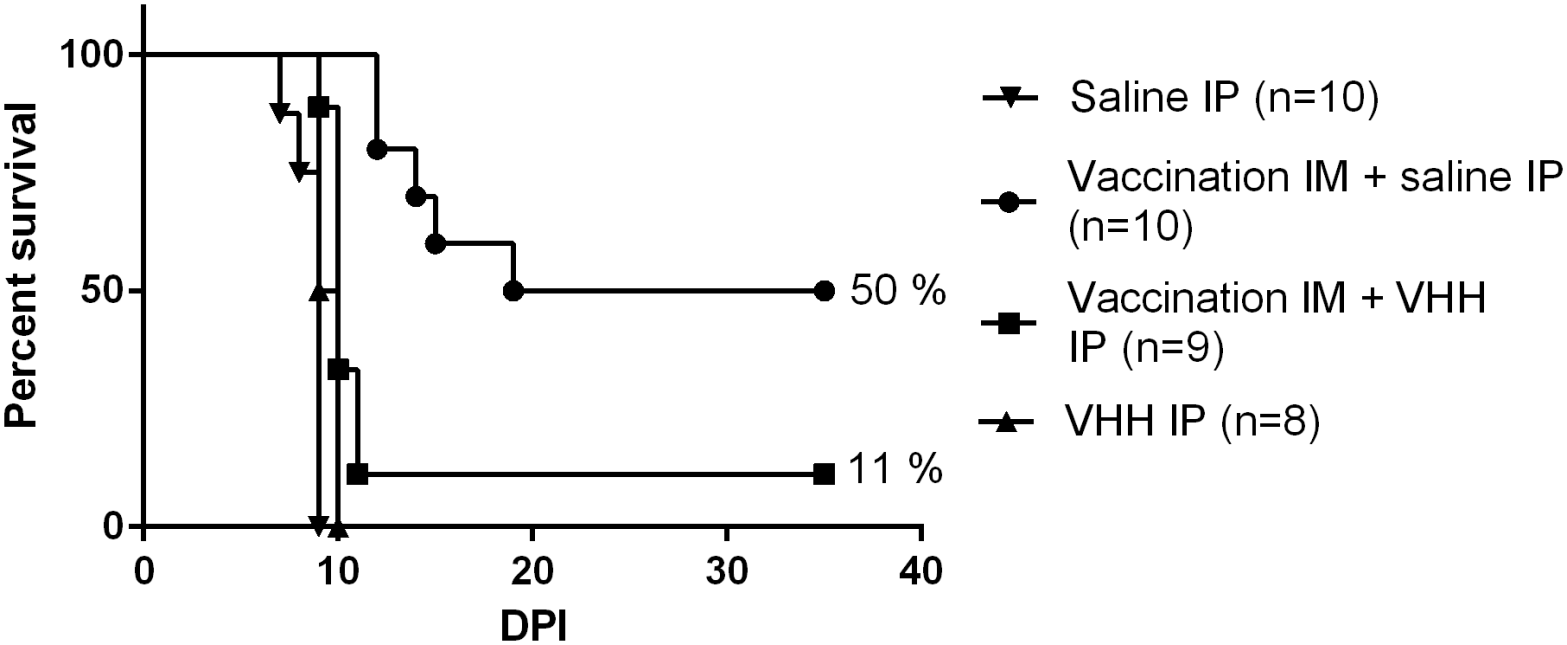
Effect of pre-exposure vaccination on survival in rabies mouse model. Mice received intramuscular (IM) vaccination at day -28 alone or in conjunction with intraperitoneal (IP) anti-rabies VHH (Rab-E8-H7-ALB11, 1.5 mg/mouse). Vaccinated mice received a booster vaccination at day -25. Control groups received a single dose of anti-rabies VHH or mock treatment (Saline) at day -28. Preventive vaccination could protect 50% of the animals from lethal infection whereas mice receiving vaccine simultaneously with anti-rabies VHH, or VHH alone, were significantly less protected from lethal disease (p<0.001).

Mice were challenged by intranasal virus inoculation 4 weeks after the start of the vaccination (day 0). Fig 2 shows the survival curves of the vaccinated and control mice. Despite the fact that all mice had high neutralizing antibody titers at the time of challenge, only 50% was protected from disease and survived the challenge. In the remaining mice disease progression was delayed (median survival time 27 days versus 9 days in control group). Disease signs in vaccinated mice were different compared to control mice, which typically develop signs of depression, such as unresponsiveness to stimuli and isolation from the group (S1 Video). The vaccinated animals remained responsive to stimuli and aware of the environment, while developing ascending paresis, starting at the hind limbs, that gradually evolved into paralysis. Eventually, mice had to be euthanized because of severe paresis and paralysis (S2 Video).

The survival of mice that received the combination regime 4 weeks before viral challenge was substantially reduced compared to the mice that received only the vaccine (11% versus 50%). The median survival time of these mice was not significantly different from the control group (10 days versus 9.5 days), despite the presence of relatively high neutralizing antibody titers at the moment of challenge. As expected, survival rates of mice that received anti-rabies VHH were comparable to the control group. The presence of the anti-rabies VHH in the circulation hence seems to reduce the vaccine efficacy. This may indicate that in absence of the virus, the binding of the anti-rabies VHH to the vaccine may interfere with the induction of an effective humoral immune response.

### 2. Post-exposure prophylactic treatment with anti-rabies VHH and vaccine

In previous *in vivo* studies, post-exposure treatment with the anti-rabies VHH one day after virus challenge was shown to provide protection from disease and death in a dose-dependent manner [15]. The same set-up was used to examine the efficacy of the combination of vaccine with a single anti-rabies VHH dose after exposure to the virus, which is the main indication for the use of vaccine in humans. Two different experiments were conducted. In a first experiment mice were treated with IP administered anti-rabies VHH (Rab-E8/H7-ALB11, 1,5 mg = 7852 IU/mouse) and IM administered vaccine (0.25 AU/mouse), twenty-four hours after challenge with a lethal rabies dose. A second vaccine dose was administered 3 days after the first. This treatment was than compared to treatment with anti-rabies VHH at the same dose or the vaccine regimen alone. The anti-rabies VHH dose was the lowest effective dose in post-exposure treatment in previous studies [15]. Similar to the pre-exposure set-up, vaccinated mice received a second vaccination 3 days after the first dose.

In the second experiment, the same vaccination schedule was applied, but instead of anti-rabies VHH, mice were treated with human rabies immune globulins (HRIG, Berirab, IP, 1 ml/mouse = 121.50 IU/mouse) at 24h after virus challenge. This is the highest volume and dose of the commercial HRIG product which could be administered to mice. Control mice were treated with HRIG alone. A schematic overview of both experiments can be found in Table 2.

**Table 2:** Set-up of the post-exposure treatment experiment and interventions in different treatment groups.

	Group	Intervention at day…			
		0	1	3	35
**Experiment 1**	**Vaccine + VHH (n = 10)**	Virus challenge	Vaccine + anti-rabies VHH	Vaccine	End observation period—euthanasia
	**Vaccine only (n = 10)**	Virus challenge	Vaccine	Vaccine	End observation period—euthanasia
	**VHH only (n = 21)**	Virus challenge	Anti-rabies VHH	Saline	End observation period—euthanasia
	**Saline (n = 7)**	Virus challenge	Saline	Saline	End observation period—euthanasia
**Experiment 2**	**Vaccine + HRIG (n = 10)**	Virus challenge	Vaccine + HRIG	Vaccine	End observation period—euthanasia
	**HRIG only (n = 7)**	Virus challenge	HRIG	Saline	End observation period—euthanasia
	**Saline (n = 10)**	Virus challenge	Saline	Saline	End observation period—euthanasia

**Fig 3:**
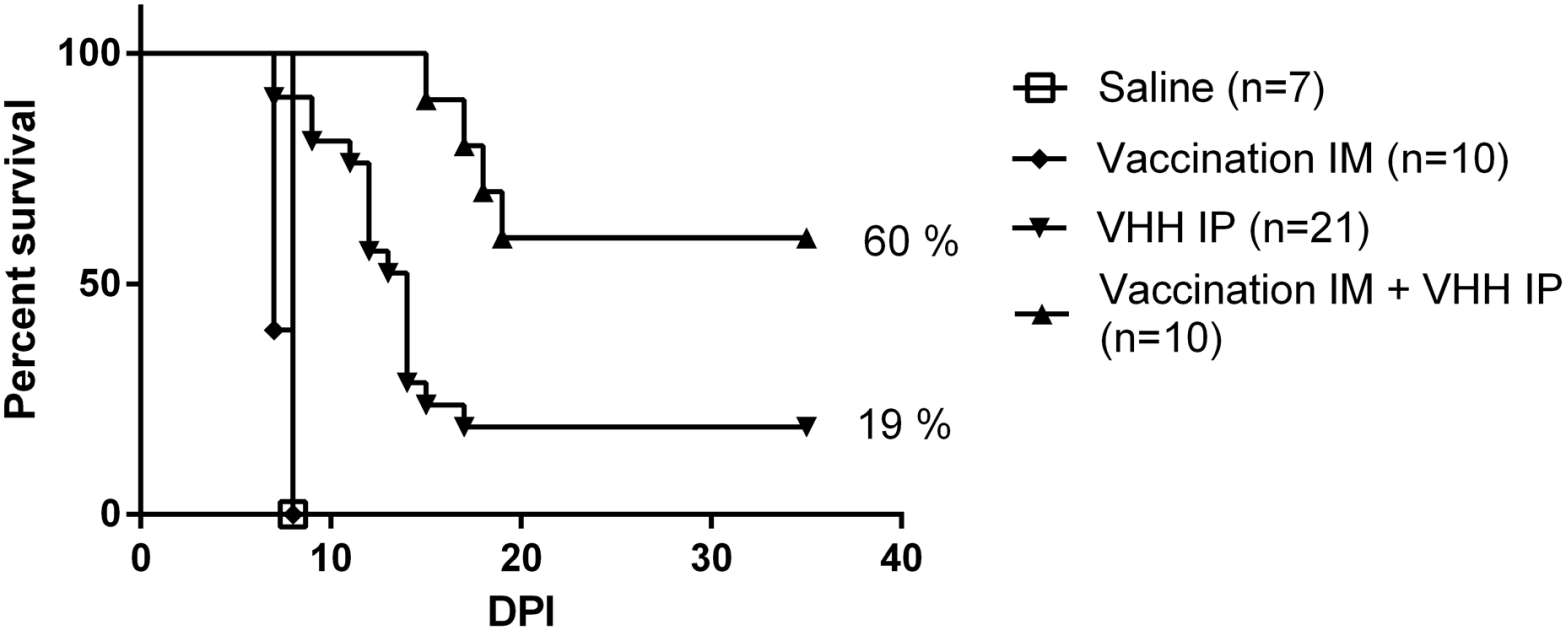
Effect of post-exposure prophylactic treatment with vaccine and anti-rabies VHH on survival in rabies mouse model. Mice were intranasally inoculated with rabies virus followed by treatment with anti-rabies VHH (IP) 24 hours later, either alone or in conjunction with vaccine (IM). Vaccinated mice received a second vaccine dose 3 days later. Control groups consisted of mice that were not treated (virus only group) or that received the vaccination regime only (vaccination group). Combined treatment with vaccine and anti-rabies VHH resulted in 60% survival, while treatment with anti-rabies VHH alone rescued 19% (p<0.01).

**Fig 4:**
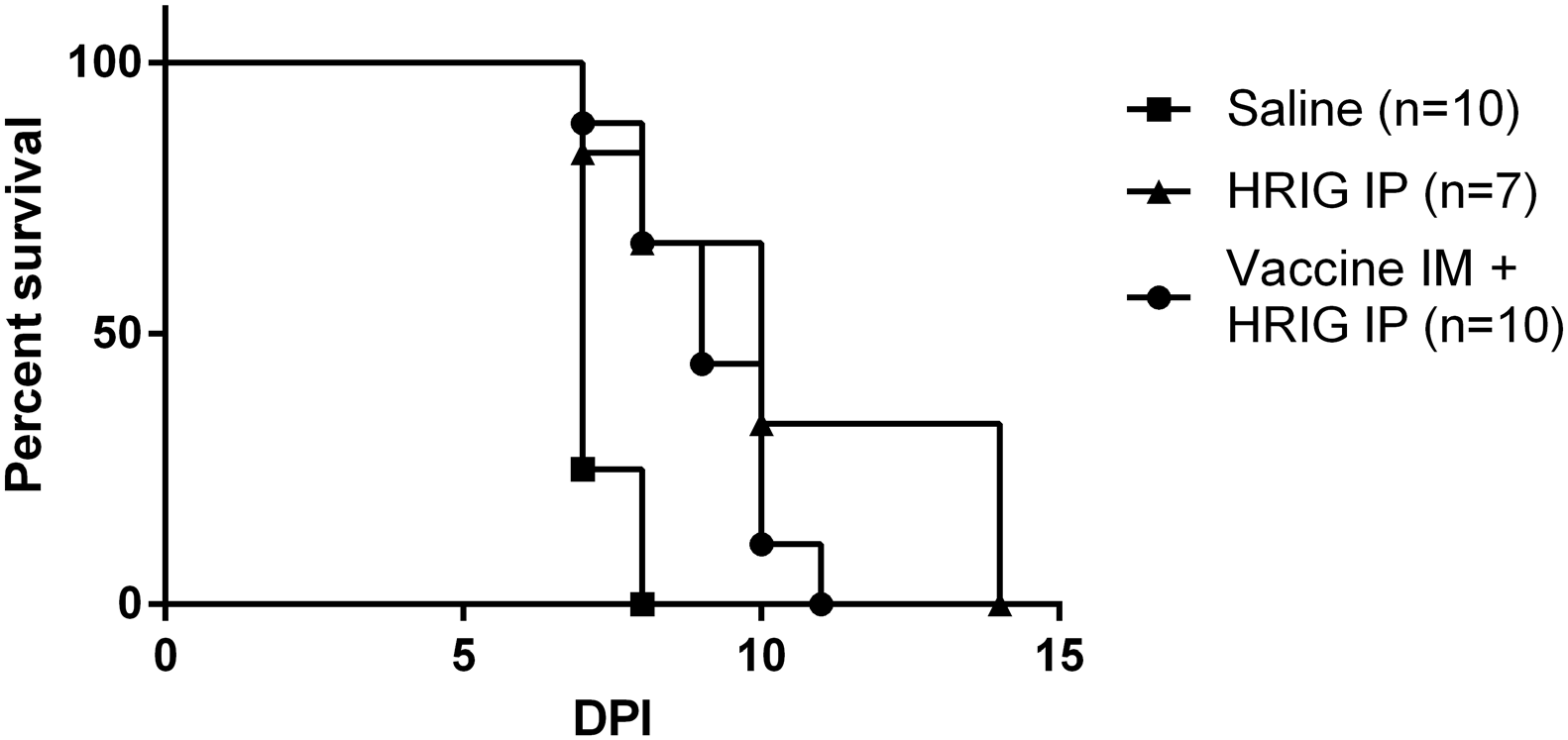
Effect of post-exposure prophylactic treatment with vaccine and human rabies immune globulins on survival in rabies mouse model. Mice were intranasally inoculated with rabies virus followed by treatment with human rabies immune globulins (HRIG) (IP) 24 hours later, either alone or in conjunction with vaccine (IM). Vaccinated mice received a second vaccine dose 3 days later. The control group consisted of mice that were not treated (virus only group). Combined treatment with vaccine and human rabies immune globulins did not differ significantly from treatment with human rabies immune globulins alone and was unable to rescue mice from lethal infection.

The survival curves of the different treatment groups in the post-exposure prophylaxis setting are depicted in Figs 3 and 4. In the post-exposure setting, the combination of vaccination with anti-rabies VHH rescued 60% of mice (Fig 3), significantly better than the treatment with anti-rabies VHH alone which rescued only 19% of mice. The vaccine by itself in the post-exposure setting did not provide any protection, and disease was similar to the control group. The median survival time was significantly longer after the combined treatment (>35 days), compared to treatment with anti-rabies VHH (14 days, p<0.01) only, vaccine only (7 days, p<0.001) or the control group (8 days, p<0.001). Mice that were treated with the combination of vaccine and HRIG did not survive challenge, similar to mice treated with HRIG alone. The median survival time of mice treated with vaccine and HRIG was 9 days and treatment with HRIG alone resulted in a median survival time of 10 days.

**Fig 5:**
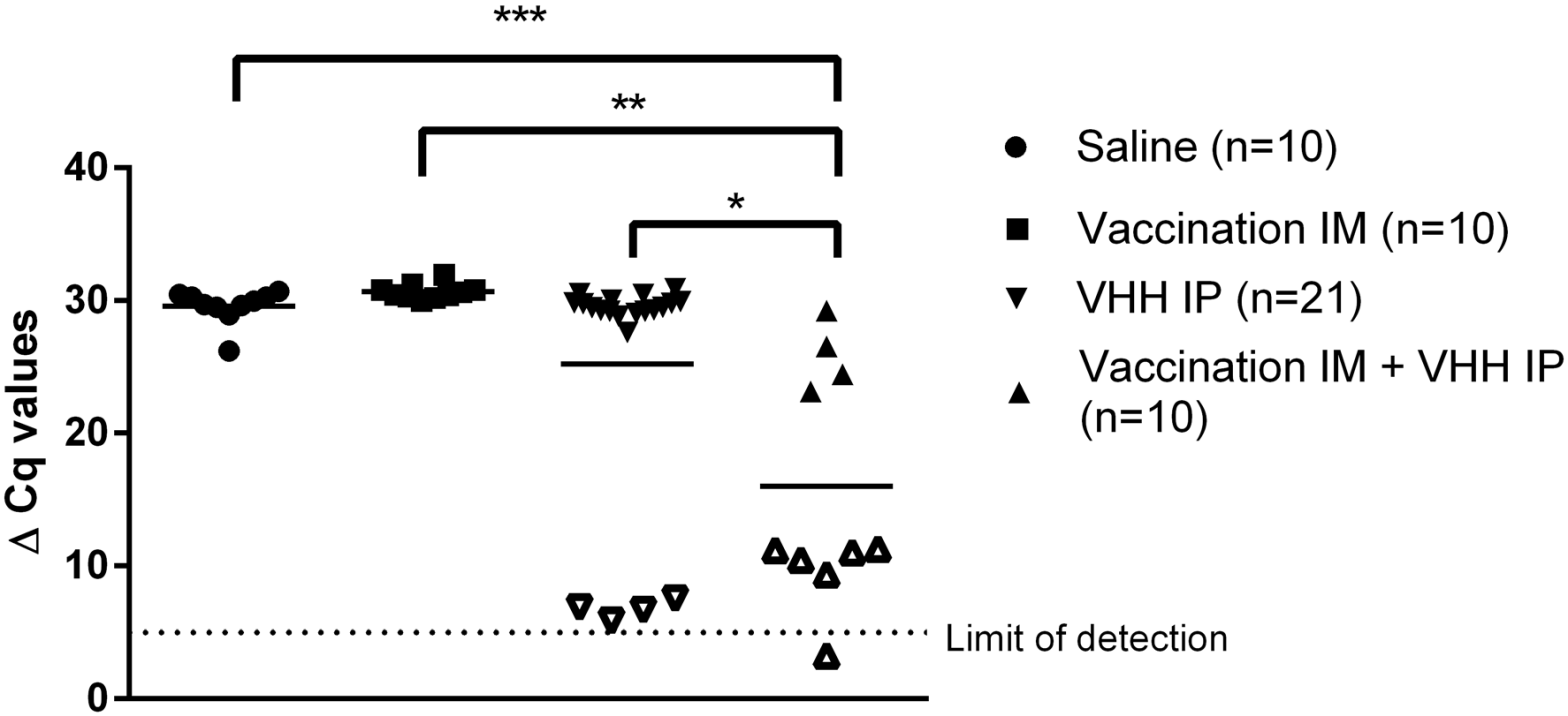
Post-exposure treatment with vaccine and anti-rabies VHH: Effect on the viral RNA load in the brain of mice. The viral load was determined at the peak of clinical symptoms in mice that developed disease (filled symbols) or at the end of the observation period (open symbols) in survivor (non-diseased) mice. The dashed line represents the limit of detection (= 5 ΔCq). Mice treated with vaccine + VHH had significantly lower viral RNA loads than naïve mice (p<0.0001), mice treated with vaccine only (p<0.001) or mice treated with VHH only (p<0.05). Viral loads of diseased mice were also lower (25.85 ΔCq) in mice that were treated with vaccine + VHH compared to naïve mice (29.58 ± 1.29 ΔCq) or treated with VHH only (29.59 ±0.76 ΔCq). Survivor mice (vaccine + VHH, VHH alone) had comparably low viral loads (3.3–11.3 ΔCq).

The viral RNA load in the brain of mice was also assessed (Fig 5). Mice that received the PEP with vaccine and anti-rabies VHH had significantly lower viral RNA loads than control mice or mice treated with anti-rabies VHH only (Fig 5).

Together these data show that in the post-exposure setting anti-rabies VHH acts synergistically with a standard vaccination regime to protect mice from disease after virus exposure.

## Discussion

Post exposure prophylaxis (PEP) for rabies consists of a combination of passive (human or equine immune globulins) and active immunisation (vaccine) soon after exposure. Anti-rabies immune globulins are expensive, scarce and often not available or affordable for people in developing countries, that are typically most at risk [24;25]. Also in Western countries, RIG are increasingly difficult to procure [26]. Cheaper and easier-to-produce alternatives are needed. Previously, we developed anti-rabies VHH (Nanobody) capable of neutralizing virus at picomolar doses *in vitro* [15]. We also showed that post-exposure treatment with anti-rabies VHH only is capable of prolonging the incubation period of the disease in a dose dependent manner. In the current study, we evaluated whether post exposure treatment with the combination of anti-rabies VHH (half-life extended Rab E8/H7-ALB11) and vaccine (Rabipur, Novartis) is better than single treatment with anti-rabies VHH or vaccine only. The combined treatment was tested using an intranasal challenge model of mice. Treatment was initiated at 24 hours after challenge.

In humans, rabies can have incubation periods as short as 4–6 days, especially if the virus is deposited in highly innervated facial tissues, as is often the case in children [27]. Failure of classic PEP is described for several cases, often with short incubation periods or when highly innervated tissues were infected, which allows quick entry of the virus in nerves [28–30]. In order for PEP to be effective, it is believed that the virus needs to be intercepted by passive or active immune effectors before invasion of the central nervous system [28]. In case of a short incubation period, with rapid invasion of the nervous system, PEP cannot intercept the virus in time to prevent brain infection.

Compared to anti-rabies VHH or vaccine alone, the combination therapy in a post-exposure setting significantly delayed the onset of disease, prolonged median survival time and decreased mortality. Sixty per cent of mice treated with anti-rabies VHH and vaccine survived the infection, in contrast to 0% with vaccine only and 19% with anti-rabies VHH only. This is in agreement with the observations from Servat *et al*., who also showed that PEP with vaccine only was unable to prevent lethal disease [31]. Post-exposure treatment with anti-rabies VHH only proved more effective than vaccine only. This partial protection is in line with studies previously described by our group [15]. We assume that the synergy between vaccine and VHH lies in the fact that anti-rabies VHH can immediately delay the spread of the virus and prolong the incubation period, which allows more (sufficient) time for the active immune response to mount and control the infection in part of the mice. Indeed, treatment with VHH prolongs the incubation period from six to ten days, and the earliest antibody and cellular immune response can be expected as soon as seven days after intramuscular vaccination with an inactivated rabies vaccine [32]. This hypothesis also explains the limited efficacy of the combined treatment with vaccine and HRIG. Indeed, in the current and a previous study [15], we found that administration of HRIG to mice after lethal challenge merely prolongs the median survival time by one or two days. This limited prolongation of the incubation period is probably not long enough to mount an effective immune response, able to control the virus infection before it becomes lethal. Our results indicate that an active antibody response was induced in all survivor mice, corresponding to low residual levels of viral RNA (ΔCq ≤10) in the brain at the endpoint measurement (35 DPI).

Pre-exposure treatment with vaccine (IM) and VHH (IP) seemed to partially reduce the immunogenicity of the vaccine, a phenomenon that is also described for the combination of RIG and vaccine [23;33;34]. Mice which received anti-rabies VHH in conjunction with vaccine developed significantly lower antibody titers 4 weeks later and were significantly less well protected against virus challenge. Indeed, whereas mice receiving vaccine only had a 50% survival rate and a delayed disease progression, only 11% of the mice treated with vaccine and anti-rabies VHH survived infection and no delay could be observed. These results were confirmed in independent experiments in which a pre-incubated mix of rabies virus and VHH was administered simultaneously at the same site (S1 Fig). Antibodies can interfere with active immunization via different mechanisms. Most of the described mechanisms are Fc dependent, like inhibition of the B-cell responses by binding to the Fc-receptor, cross-linking of the B-cell receptor and the complement system, or antigen removal by macrophages [35]. Only humoral, and not cellular, immune responses seem to be affected by the presence of specific antibodies [36]. Since the used anti-rabies VHH is not a full antibody and lacks the Fc domain, it is unlikely that these mechanisms are involved [37]. The half-life extended anti-rabies VHH can interact with the neonatal Fc receptor through the intermediate of albumin, but it remains an unlikely mechanism since the non-HLE anti-rabies VHH, lacking an albumin-binding VHH component showed similar reduction of the vaccine efficacy (S1 Fig). Therefore a likely mechanism could be epitope masking. By binding to the surface glycoproteins of the inactivated vaccine virus, the anti-rabies VHH might shield recognition of the epitopes by the immune system [36]. The fact that the combination of anti-rabies VHH with vaccine still proved superior in PEP, argues for the relative importance of immediate passive immunisation in PEP, especially when the virus has easy access to nerves or neuronal cells.

Pre-exposure vaccination offered only partial protection upon intranasal virus challenge (50% survivors). Half of the mice that were actively immunized with (inactivated) vaccine, both at 28 and 25 days before challenge, still developed lethal brain infection. This incomplete protection, even with high antigenic doses (2 x 0.25 AU/mouse), is also described by other researchers, using similar models [18]. Nevertheless, the applied vaccine schedule resulted in clear seroconversion of all mice, with virus-neutralizing serum titers well above the protective threshold of 0.5 IU/ml (range 6.01–18.04 IU/ml) at the moment of challenge. Moreover, the challenge occurred at four weeks after the first vaccine administration, at the moment when the peak serological response can be expected [38;39]. The height of the neutralizing antibody titer in vaccinated mice did not correspond to the level of protection upon challenge. Some mice with titers up to 20 IU/ml still developed lethal disease.

The incomplete protection in the post-exposure setting may be explained by the aggressive nature of the used intranasal challenge model, in which virus is inoculated directly on a site that contains a high concentration of olfactory neuronal cells, providing a direct portal of entry to the central nervous system. In earlier studies we found spread of the virus in the olfactory bulbs of the brain already at the first day after inoculation [15]. Once inside the central nervous system, the virus is protected from several systemic immune effectors, which may limit the protection by the vaccine [40;41]. We therefore assume that the mice that survived the challenge after preventive vaccination or PEP with anti-rabies VHH and vaccine were able to develop a cellular immune response, capable of controlling the infection in the brain.

The intranasal challenge model is our preferred experimental model because of the high reproducibility, practicability, safety and animal wellbeing issues [16]. It may be that in an infection model with a longer incubation period and a more pronounced phase of peripheral virus replication in non-neuronal cells, preventive vaccination would be more effective, since vaccine-induced antibodies might be more effective to intercept virus spread between non-neuronal and neuronal cells. In our hands, intramuscular inoculation of rabies virus requires unnaturally high levels of virus in the inoculum (>10^5−6^ CCID_50_) and yields variable inter-assay results, limiting its use for experimental comparison of intervention strategies [16].

Another remarkable finding was the different clinical picture observed depending on the vaccination status of the mouse prior to virus challenge. Naïve mice typically showed signs of depression, such as isolation from the group, inactivity and unresponsiveness to stimulation. In contrast, pre-immunised mice remained alert and vivid, but developed ascending paresis, resulting in paralysis of all limbs, requiring euthanasia. Vaccinated mice developed disease after a longer incubation period (13.7 instead of 9 days) and had a longer morbidity period (3 instead of 1.5 days), which resulted in a longer median survival time (27 instead of 9 days), compared to naïve mice. They also had lower viral loads in the brain at the peak of disease. The vaccine-induced immune response thus had a clear effect on pathogenesis and symptomatology. Iwasaki *et al*. also found that the host immune response has a clear impact on the development of, what they refer to as, either “encephalitic” or “paralytic” disease in mice. Rabies virus challenge in immunocompetent mice resulted in “paralytic disease”, with relatively low viral loads and a high extent of inflammation and damage in the brain. The same challenge in cyclophosphamide-treated mice resulted in the absence of an immune response and “encephalitic disease”, with severe general depression, only minor paralysis, high viral loads, and less neuronal cell damage [42]. In our study, the pre-immunized mice developed a disease pattern similar to the immunocompetent mice of Iwasaki *et al*., whereas the naïve mice evolved comparably to the cyclophosphamide-treated mice. In human cases, the average survival time of paralytic rabies is twice as long, compared to the encephalitic (furious) form [42]. Patients with paralytic rabies typically remain fully conscious, while developing ascending motor weakness [43]. Also in dogs, paralytic rabies is associated with reduced viral load and more prominent inflammation [44]. Our observations further add to the evidence that paralytic rabies may be caused by an immuno(patho)logical response of the host to the virus infection.

In humans, passive immunisation with anti-rabies antibodies is expected to bridge the immunity gap between virus exposure and onset of the active antibody production induced by the vaccine. The half-life extension of the anti-rabies VHH is based on the addition an anti-albumin VHH component. In mice, addition of anti-albumin VHH extends the half-life to 0.5–1.9 days [15], while in humans it is extended up to 10–20 days [45]. It would therefore be feasible to formulate and dose anti-rabies VHH for humans to obtain protective levels (> 0.50 IU/ml) in the blood for 14 days, which would be sufficient for the active immune response to take over. Compared to (human) rabies immune globulins (150 IU/ml), VHH can be produced and formulated at very high potencies (>6000 IU/ml). WHO recommends that rabies immune globulins are administered locally into the wound, however, due to the limited potency per ml of the rabies immune globulins, this is not possible for small wounds or injuries to nose, fingers or toes as it can cause compartment syndrome. VHH formulations containing high potencies per ml could overcome this problem and would be more suited for infiltration of the whole dose into small body parts.

These results provide evidence for the possible use of anti-rabies VHH together with vaccine for post exposure prophylaxis of rabies. Early treatment with anti-rabies VHH can delay the incubation period of the disease, which allows more time for the vaccine-induced immunity to control the infection. The ease of production and high thermal stability of VHH are important advantages over the currently used anti-rabies immune globulins.

## Supporting Information

S1 Video Typical disease symptoms of rabies in mice. This video shows typical symptoms of late stage rabies disease in mice. Animals show signs of apathy and depression.
(AVI)

S2 Video Disease symptoms of rabies in mice with pre-existing immunity. This video shows the contrasting disease symptoms in mice with pre-existing immunity. In contrast to “naïve” mice, mice shows symptoms of incoordination rather than apathy. In these animals disease progression is slower than in naïve mice.
(AVI)

S1 Fig Rabies neutralizing activity in the blood as measured by RFFIT, following intraperitoneal administration (IP) of a pre-incubated mix of HLE VHH or non-HLE VHH and vaccine at day -28 and day -14. The control group consisted of mice receiving the vaccine without VHH. Blood was collected at day -28 (prior to vaccination and VHH administration), day -14 and day 0. Mice that received rabies vaccination had high antibody titers from day -14 onwards whereas mice that received the pre-incubated mix of HLE VHH + vaccine or non-HLE VHH + vaccine had significantly lower antibody titers on both days (*** p<0.0001, ** p<0.005, * p<0.01). Error bars represent the standard deviation.
(TIF)
