# Supplementary figures and images for: Post-exposure Treatment with Anti-rabies VHH and Vaccine Significantly Improves Protection of Mice from Lethal Rabies Infection
Source: PLoS Negl Trop Dis. 2016 Aug 2;10(8):e0004902. doi: 10.1371/journal.pntd.0004902 (PMC4970669; doi:10.1371/journal.pntd.0004902)

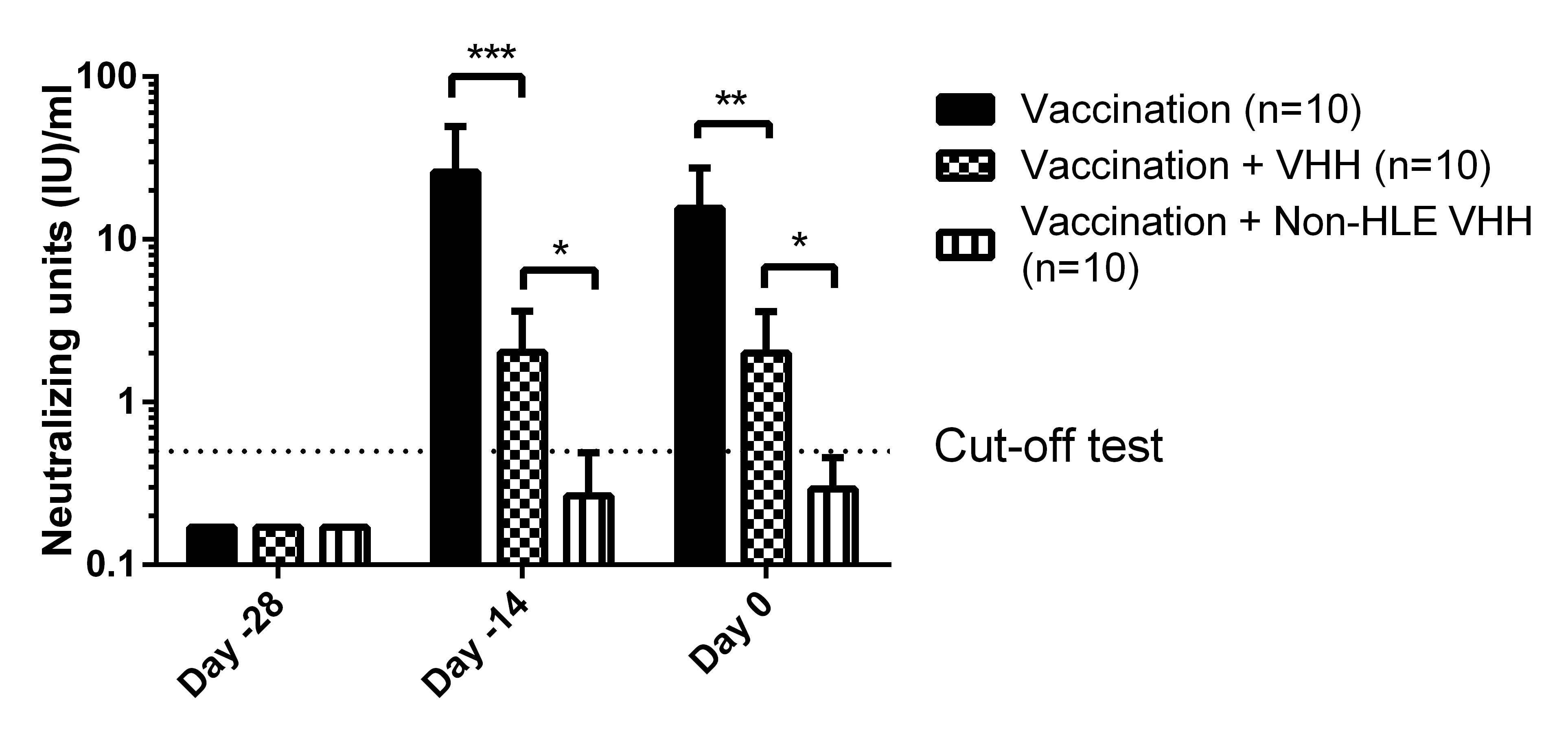

Supplement: S1 Fig — The control group consisted of mice receiving the vaccine without VHH. Blood was collected at day -28 (prior to vaccination and VHH administration), day -14 and day 0. Mice that received rabies vaccination had high antibody titers from day -14 onwards whereas mice that received the pre-incubated mix of HLE VHH + vaccine or non-HLE VHH + vaccine had significantly lower antibody titers on both days (*** p<0.0001, ** p<0.005, * p<0.01). Error bars represent the standard deviation. (TIF) [file pntd.0004902.s003.tif]
